# Supplementary material for: Kineococcus vitellinus sp. nov., Kineococcus indalonis sp. nov. and Kineococcus siccus sp. nov., Isolated Nearby the Tabernas Desert (Almería, Spain)
Source: Microorganisms. 2020 Oct 7;8(10):1547. doi: 10.3390/microorganisms8101547 (PMC7601052; doi:10.3390/microorganisms8101547)
Supplement: Supplementary file 1 [file microorganisms-08-01547-s001.pdf]

Supplementary Material

Kineococcus vitellinus sp. nov., *Kineococcus indalonis* sp. nov. and *Kineococcus siccus* sp. nov., Isolated Nearby the Tabernas Desert (Almería, Spain)

Esther Molina-Menor [1], Helena Gimeno-Valero [2], Javier Pascual [2], Juli Peretó [1,2,3], Manuel Porcar [1,2,] *

[1] Institute for Integrative Systems Biology I2SysBio (Universitat de València-CSIC), Calle del Catedràtic Agustin Escardino Benlloch 9, 46980 Paterna, Spain

[2] Darwin Bioprospecting Excellence SL. Parc Científic Universitat de València, Calle del Catedràtic Agustin Escardino Benlloch 9, 46980 Paterna, Spain

[3] Departament de Bioquimica i Biologia Molecular, Universitat de València, Calle del Dr. Moliner 50, 46100 Burjassot, Spain

* Correspondence: manuel.porcar@uv.es

Table S1. Carbon source utilization comparison using GENIII MicroPlates of strains T13^{T}, T90^{T}, R8^{T} and the type strains of closely related *Kineococcus* species. Strains: 1, T13^{T}; 2, T90^{T}; 3, R8^{T}; 4, *Kineococcus radiotolerans* DSM 14245^{T}; 5, *Kineococcus aureolus* DSM 102158^{T}; 6, *Kineococcus aurantiacus* DSM 7487^{T}; 7, *Kineococcus gypseus* DSM 27627^{T}; 8, *Kineococcus mangrovi* NBRC 110933^{T}; 9, *Kineococcus gynurae* NBRC 103943^{T}. Data for reference strains were obtained in the present study. +, positive; -, negative. All strains are positive for D-turanose. All strains are negative for p-hydroxy-phenylacetic acid, N-acetyl neuraminic acid and α-hydroxy-butyric acid.

**Characteristic**	**1**	**2**	**3**	**4**	**5**	**6**	**7**	**8**	**9**
D-Raffinose	+	+	-	+	+	-	-	+	-
α-D-Glucose	+	+	-	+	+	+	+	+	-
D-Sorbitol	+	+	-	+	+	+	-	+	-
Gelatine	-	+	-	-	+	+	-	+	-
Pectin	+	+	+	+	+	-	+	+	-
p-Hydroxy-phenylacetic acid	-	-	-	-	-	-	-	-	-
Tween 40	+	+	+	-	+	+	+	+	-
Dextrin	+	+	+	+	-	+	+	+	+
α-D-Lactose	+	+	-	+	-	-	+	+	-
D-Mannose	+	+	-	+	-	-	+	+	-
D-Mannitol	+	+	-	+	-	-	-	+	-
Glycyl-L-proline	-	+	-	-	-	-	-	-	-
D-Galacturonic acid	-	+	-	-	+	-	-	+	-
Methyl pyruvate	-	-	-	-	+	-	-	-	-
γ-Amino-butyric acid	-	+	-	-	-	-	-	-	-
D-Maltose	+	+	-	+	+	-	+	+	+
D-Melibiose	+	+	-	+	+	-	-	+	+
D-Fructose	+	+	+	+	+	-	+	+	-
D-Arabitol	-	+	+	+	+	-	-	+	-
L-Alanine	+	+	+	+	+	-	-	-	-
L-Galactonic acid lactone	-	+	-	-	-	-	-	-	-
D-Lactic acid methyl ester	-	+	-	-	-	-	-	-	-
α-Hydroxy-butyric acid	-	-	-	-	-	-	-	-	-
D-Trehalose	+	+	-	+	+	-	-	+	-
β-Methyl-D-glucoside	+	+	-	+	+	-	+	+	-
D-Galactose	+	+	+	+	+	-	+	+	-
myo-Inositol	+	+	-	+	+	-	-	+	-
L-Arginine	-	+	-	-	-	-	-	-	-
D-Gluconic acid	-	+	+	+	+	+	+	+	+
L-Lactic acid	-	-	+	-	+	-	-	-	-
β-Hydroxy-D,L-butyric acid	-	+	-	-	-	-	-	-	-
D-Cellobiose	+	+	+	+	+	+	+	+	-
D-Salicin	+	+	-	+	+	-	+	+	-
3-Methyl-D-glucoside	-	+	+	-	-	-	-	+	-
Glycerol	+	+	+	+	+	+	+	+	-
L-Aspartic acid	-	+	-	-	-	-	-	-	-
D-Glucuronic acid	-	+	+	-	+	-	-	-	-
Citric acid	-	+	-	-	-	-	-	-	-
α-Keto-butyric acid	-	+	-	+	-	-	-	-	-
Gentiobiose	+	+	+	+	+	-	+	+	-
N-acetyl-D-glucosamine	+	+	-	-	-	-	-	-	-

D-Fucose	-	+	-	+	-	-	+	-	-
D-Glucose-6-PO_4	-	+	-	+	-	+	-	-	+
L-Glutamic acid	-	+	-	+	+	-	-	+	-
Glucuronamide	+	+	+	-	-	+	+	-	-
α-Keto-glutaric acid	-	+	-	-	+	-	-	-	-
Acetoacetic acid	+	+	+	+	+	+	+	+	-
Sucrose	+	+	-	+	+	+	+	+	-
N-Acetyl- β-D-mannosamine	-	+	-	-	-	-	-	-	-
L-Fucose	-	+	+	+	+	-	-	+	-
D-Fructose-6-PO_4	+	+	+	-	-	+	+	-	+
L-Histidine	-	+	-	-	-	-	-	-	-
Mucic acid	-	+	+	-	+	-	-	-	-
D-Malic acid	-	+	+	+	+	-	-	-	-
Propionic acid	-	+	-	-	+	-	-	-	-
D-Turanose	+	+	+	+	+	+	+	+	+
N-acetyl-D-galactosamine	-	+	-	-	-	-	-	-	-
L-Rhamnose	+	+	-	+	+	-	-	+	-
D-Aspartic acid	-	+	+	-	-	-	-	-	-
L-Pyroglutamic acid	+	+	-	+	+	-	-	-	-
Quinic acid	-	-	+	-	-	-	-	-	-
L-Malic acid	-	+	+	+	+	-	-	+	-
Acetic acid	-	+	+	+	+	-	-	+	-
Stachyose	+	+	-	+	+	-	-	+	-
N-Acetyl neuraminic acid	-	-	-	-	-	-	-	-	-
Inosine	-	-	-	-	+	-	-	-	-
D-Serine	-	+	+	-	-	-	-	-	-
L-Serine	+	+	-	-	-	-	-	-	-
D-Saccharic acid	-	-	-	-	+	-	-	-	-
Bromo-succinic acid	-	+	-	-	+	-	-	-	-
Formic acid	-	-	-	-	-	-	+	-	-

Figure S1. Cell morphology of strains T13^{T} (A, B), T90^{T} (C, D) and R8^{T} (E, F) under the optical microscope at 100× magnification. Cells were obtained from an overnight culture in trypticase soy broth liquid media at 30 °C and stained with crystal violet glass. Size bars correspond to 3 μm.

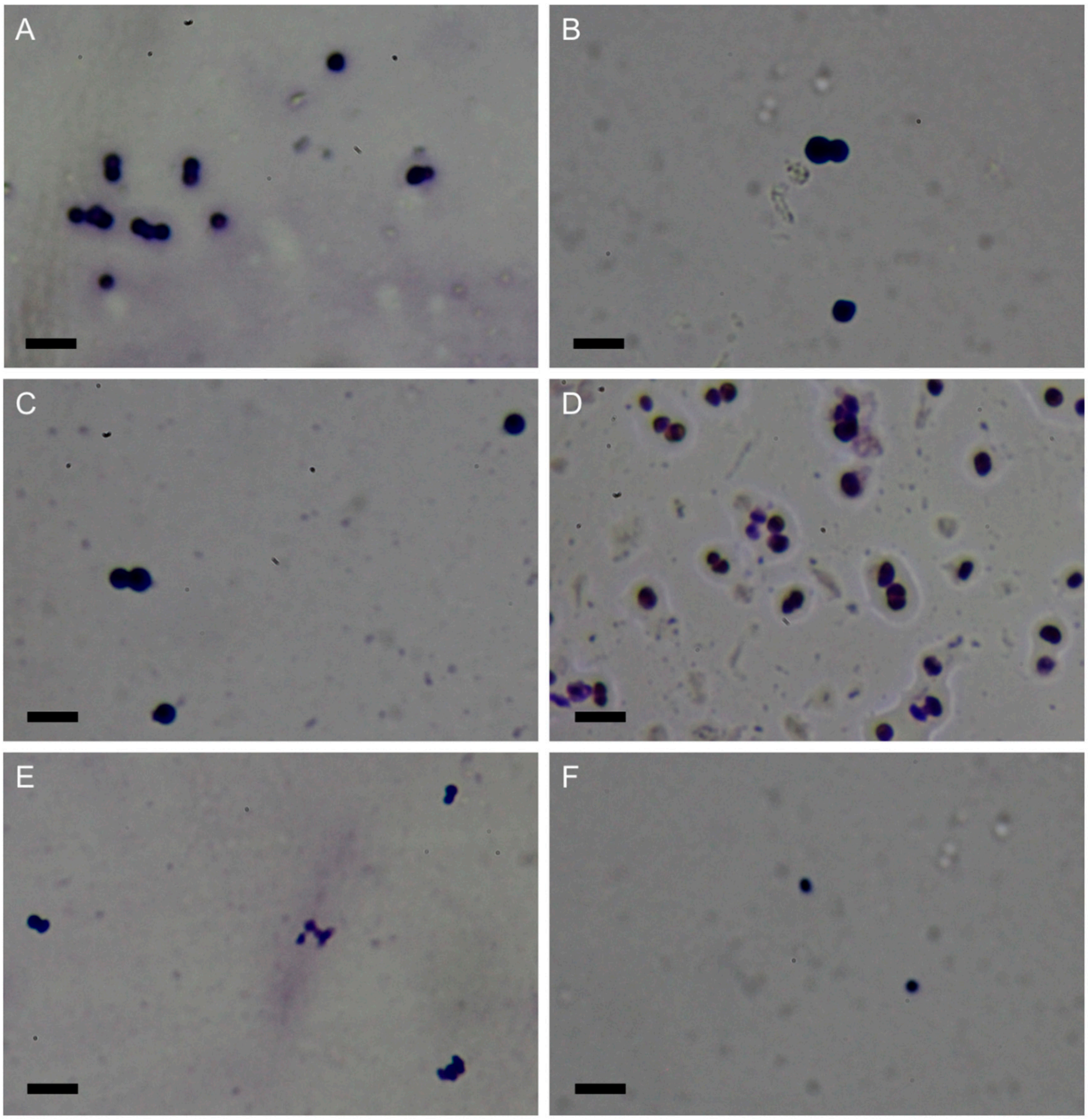

Supplementary Material

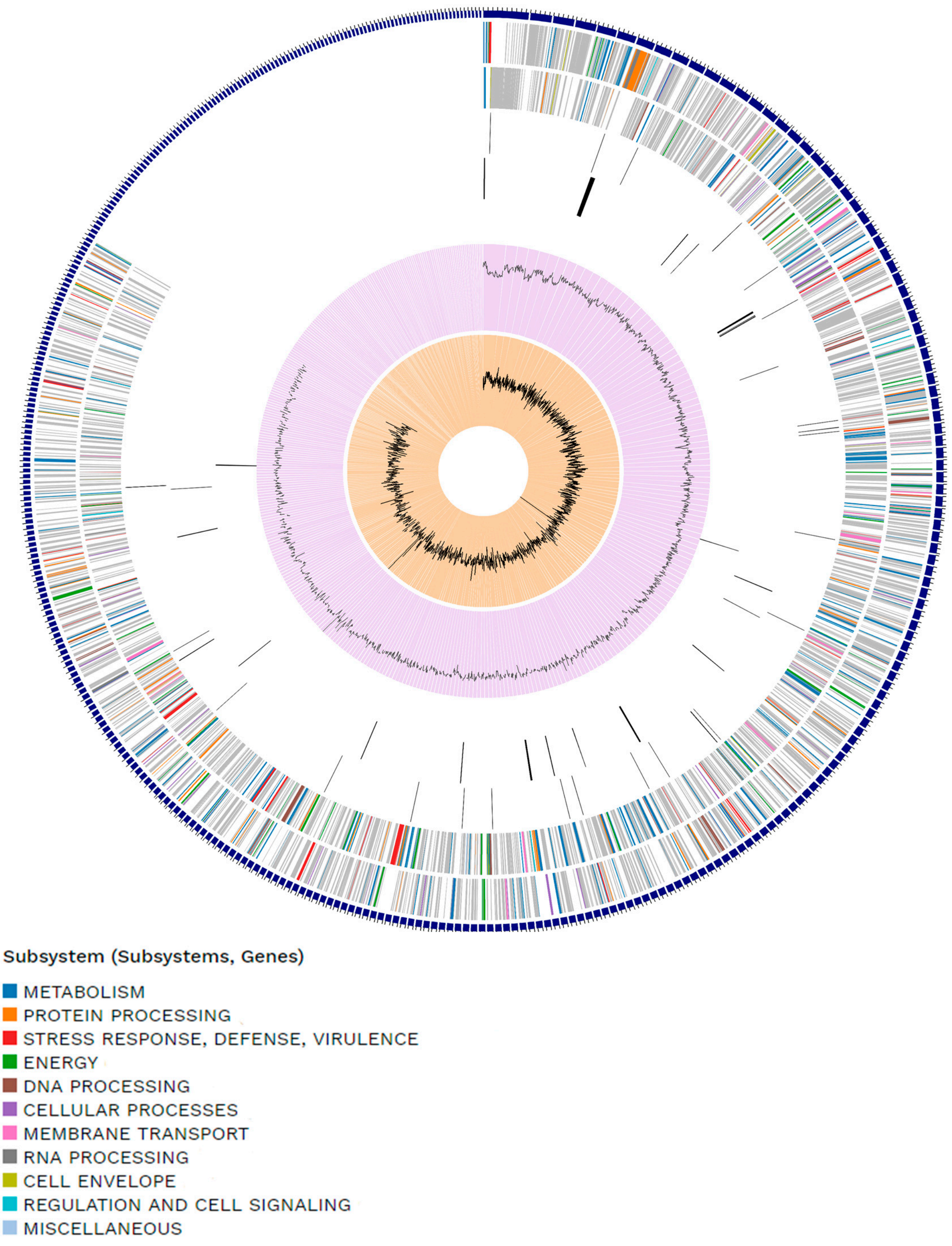

Figure S2. *Kineococcus vitellinus* sp. nov. T13^{T} circular genomic map.

Supplementary Material

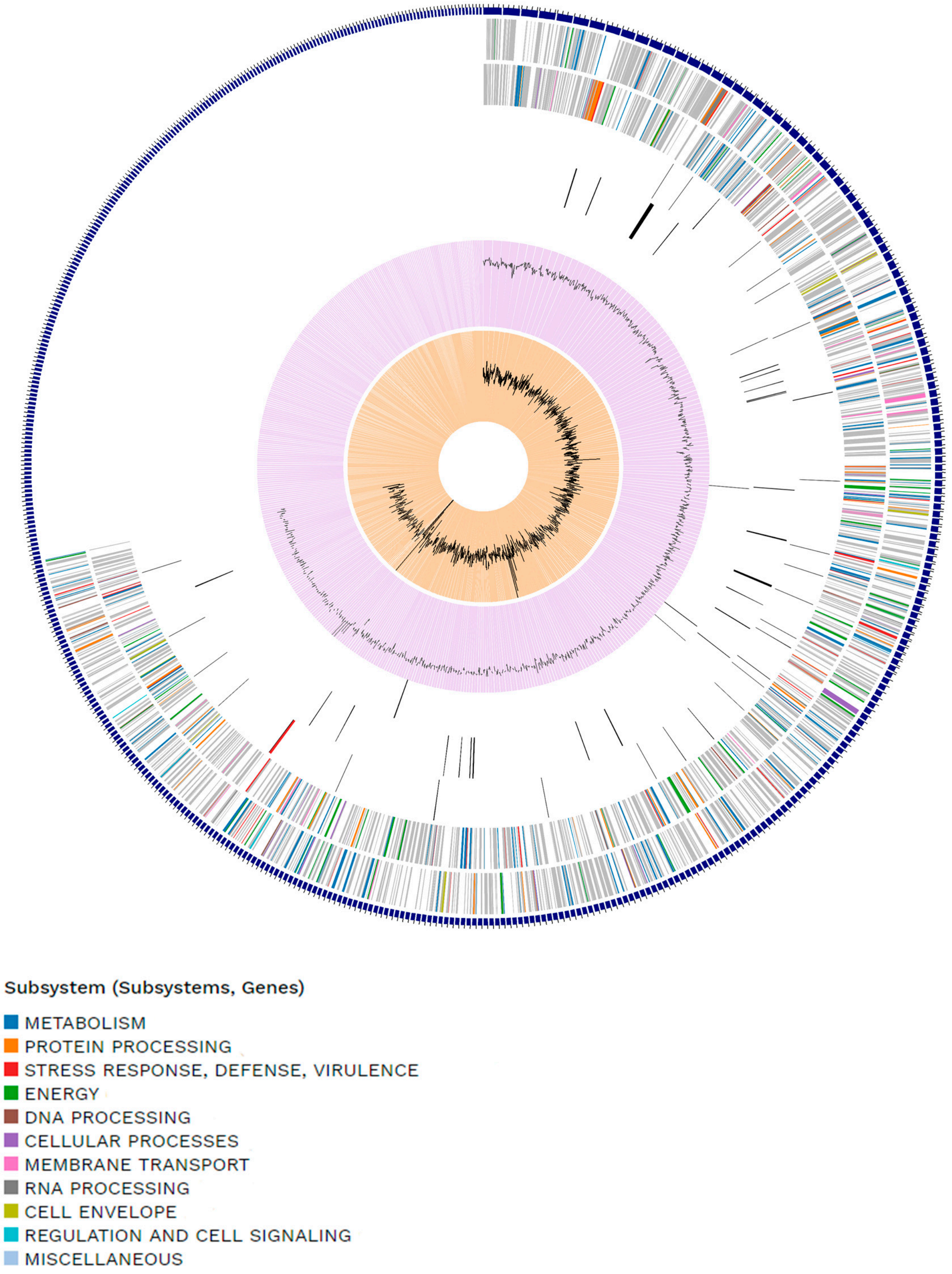

Figure S3. *Kineococcus indalonis* sp. nov. T90^{T} circular genomic map.

Supplementary Material

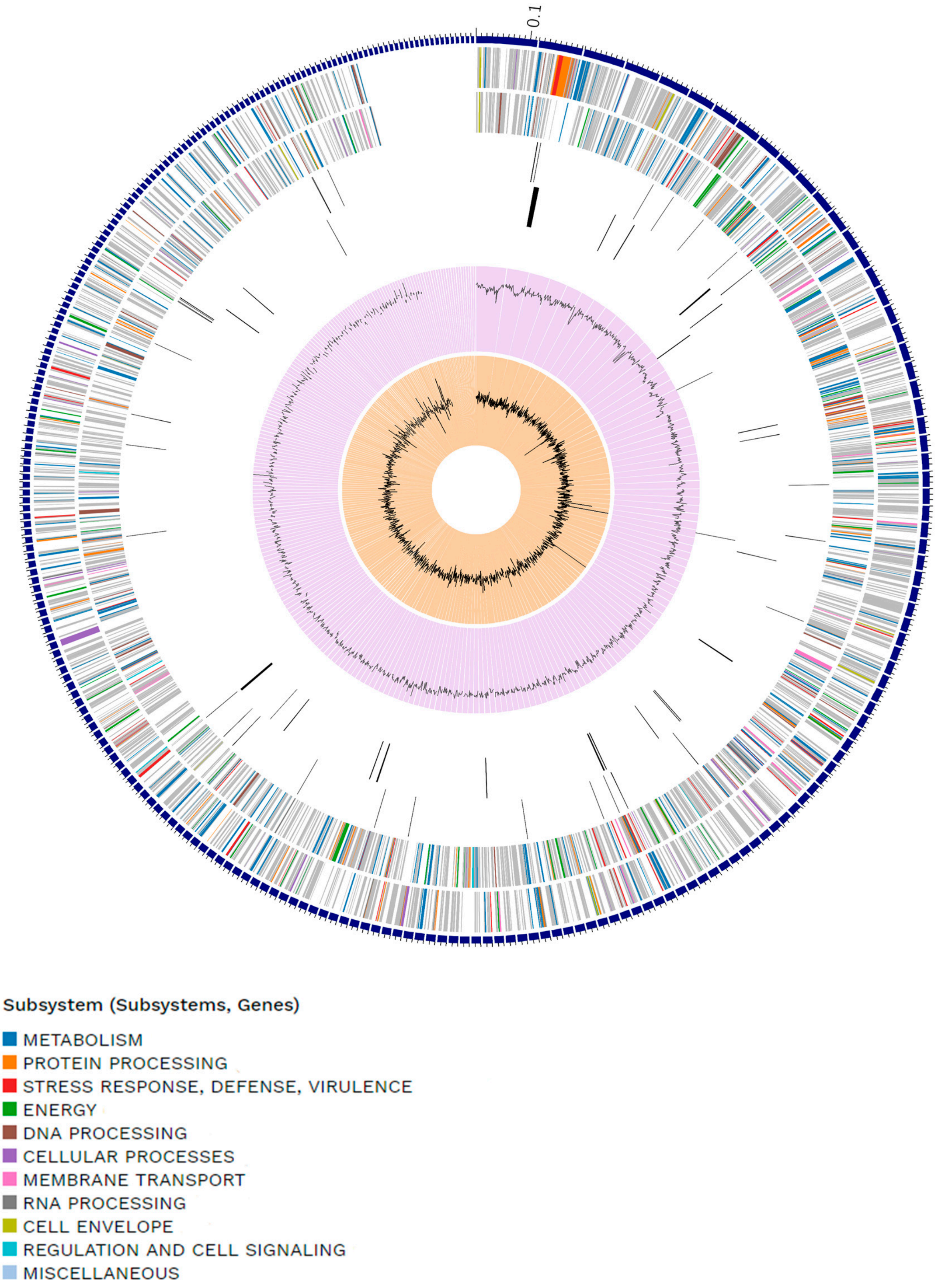

Figure S4. *Kineococcus siccus* sp. nov. R8^T circular genomic map.
